# Roth Net-Assisted Endoscopic-Guided Manometry Catheter Placement

**DOI:** 10.7759/cureus.10063

**Published:** 2020-08-26

**Authors:** Dhineshreddy Gurala, Jobin Philipose, Abhishek D Polavarapu, Youssef El Douaihy, Stephen M Mulrooney

**Affiliations:** 1 Internal Medicine, Staten Island University Hospital - Northwell Health, New York, USA; 2 Gastroenterology and Hepatology, Staten Island University Hospital - Northwell Health, New York, USA; 3 Gastroenterology, Staten Island University Hospital - Northwell Health, New York, USA

**Keywords:** manometry, roth net, endoscopy, dysphagia, achalasia, motility

## Abstract

High-resolution esophageal manometry (HRM) has become the gold standard to diagnose esophageal motility disorders. Usually, this procedure is performed by introducing the catheter, which has pressure sensors, into the esophagus and proximal stomach via the nares. Repeated coiling of the catheter and inability to pass through the gastroesophageal junction (GEJ) are common challenges encountered. Endoscopy-guided placement of the catheter can overcome these difficulties. However, sometimes even with the use of endoscopy, it is difficult to advance catheter due to anatomical variants. The extreme fragility of the catheter and sensors and the high cost of this reusable device precludes the use of biopsy forceps or snare to advance the catheter. There is no literature on using accessories during endoscopy in case of difficult placement under direct visualization. We report a unique case of using Roth Net via the suction channel to advance esophageal manometry catheter into the stomach by using endoscopy.

## Introduction

Esophageal manometry has been the method of choice for evaluation of esophageal motility disorders and also useful in the evaluation of gastroesophageal reflux disease (GERD) or non-cardiac chest pain [1]. Conventional esophageal manometry has been state of the art till the 1990s. The invention of high-resolution esophageal manometry (HRM) in 1990 led esophageal evaluation to a new horizon. HRM combined with esophageal pressure topography (EPT) significantly increased the quality of pressure sensing and analysis of data as a topographic plot on the monitor. Usually, this procedure is performed by introducing the catheter and advance through the esophagus into the stomach via the nares in an awake patient. After achieving successful cannulation of the catheter, the patient is asked to take 10 swallows of 5 cc water followed by measurement of pressures and analysis of data. Any changes in consistency and quantity of swallows can affect manometry results [2,3]. In prior studies, 21% of HRM studies were technically imperfect, and 29% of those were due to the inability to traverse through lower esophageal sphincter (LES) and 12% due to failure to complete a minimum number of swallows [1]. These difficulties were circumvented by endoscopy-guided placement of manometry catheter. However, even with endoscopy due to repeated coiling of the catheter or due to anatomical variants, cannulation is difficult and prone to damaging the catheter/sensors, which precludes the use of accessories to smoothen the process and to prevent damage to the catheter. Herein we report a unique case of difficult placement of manometry catheter under endoscopy guidance, which was completed successfully by using Roth Net.

## Case presentation

**Figure 1:**
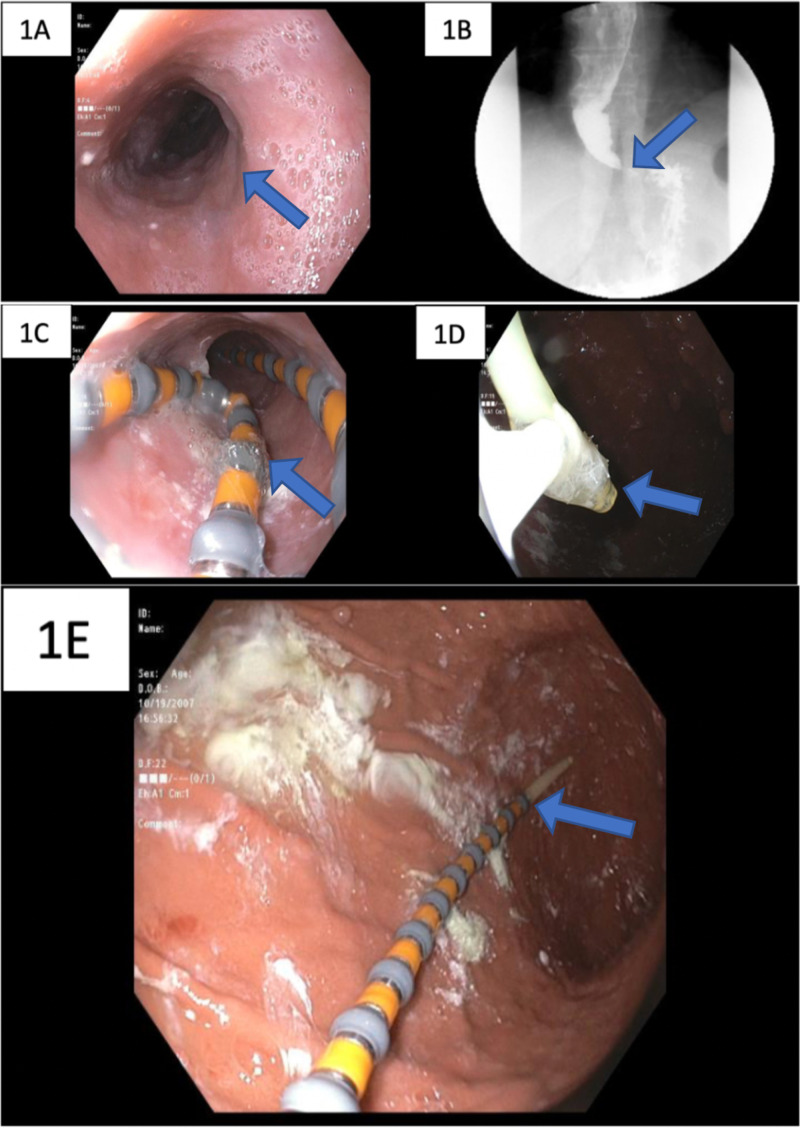
(A) Esophagogastroduodenoscopy (EGD) showing esophageal dilatation. (B) Barium esophagogram showing dilated esophagus with distal spasm (blue arrow). (C) Coiling of manometry catheter in the esophagus. (D) Tip of the manometry catheter was captured by the Roth Net. (E) Successful placement of the catheter in the antrum of the stomach.

A 59-year-old female with a history of acid reflux and diabetes, presented with dysphagia to solids and liquids for the last six months with progressive inability to swallow any food associated with weight loss of 6 pounds and loss of appetite. She denied any abdominal pain, nausea, vomiting, hematemesis, melena, hematochezia, or a family history of gastrointestinal malignancy. Vital signs at the time of admission were normal. Physical examination was negative for any abdominal distension or tenderness. Initial esophagogastroduodenoscopy (EGD) showed esophageal dilatation without any intraluminal mass, as shown in Figure 1A. Barium esophagogram showed mildly dilated esophagus and little to no peristaltic activity (Figure 1B). Due to the inability to advance the manometry catheter without sedation, she was scheduled for endoscopic-guided placement. During EGD, at gastroesophageal junction (GEJ), some resistance was met but without any luminal abnormality in the mucosa. After completion of EGD, the manometry probe was introduced via the nares and advanced under endoscopic guidance, but the catheter repeatedly coiled in the oropharynx (Figure 1C) and mid esophagus. The catheter was advanced and withdrawn to relive coiling, but still, the tip of the catheter couldn’t be advanced beyond the GEJ. A Roth Net was introduced through the suction channel of the endoscope. The motility catheter tip was gently captured with the Roth Net (Figure 1D) and guided through LES and advanced into the antrum of the stomach (Figure 1E).

**Figure 2:**
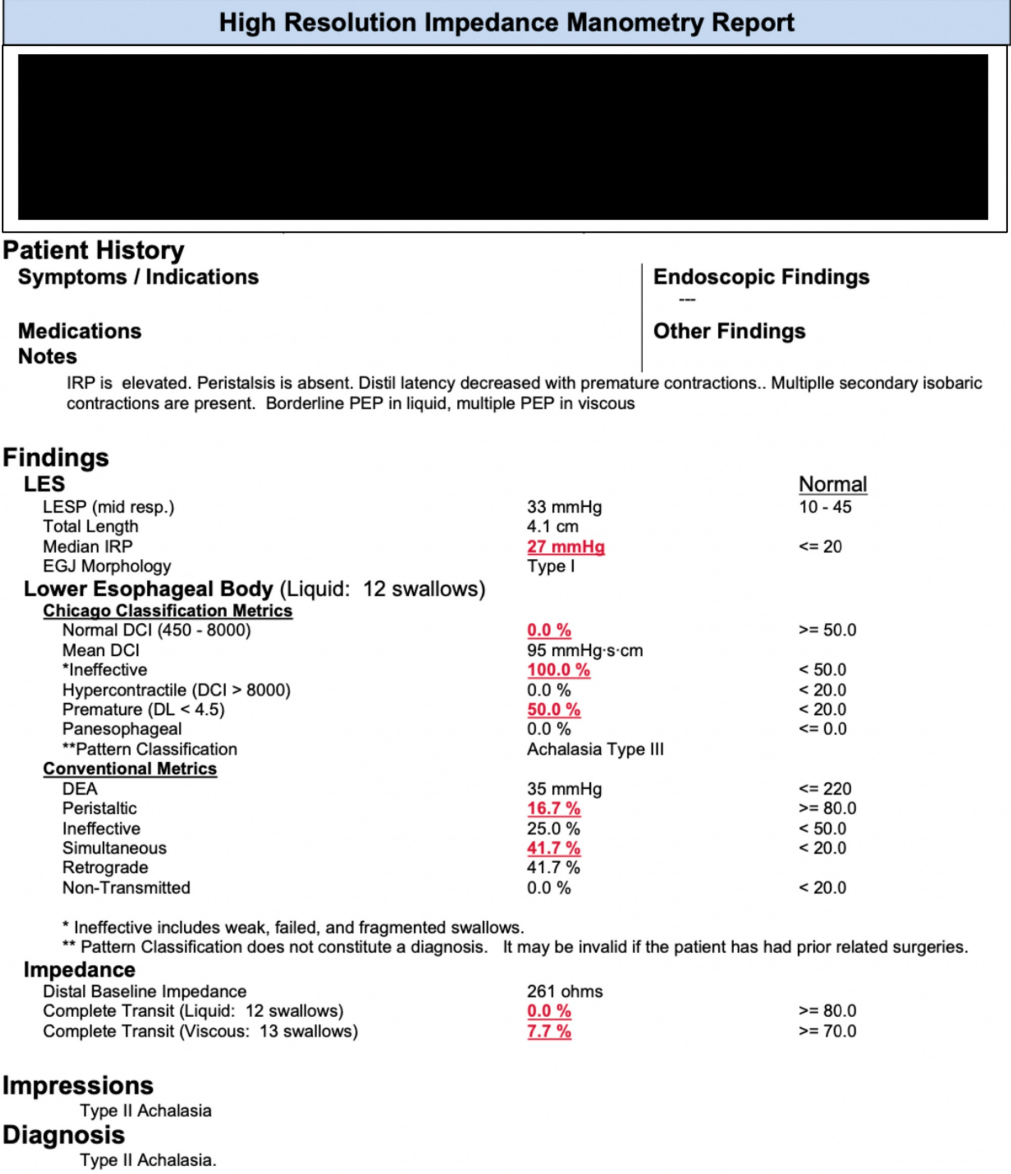
Manometry report. IRP: Integrated relaxation pressure; LES: Lower esophageal sphincter; IRP: Integrated relaxation pressure; EGJ: Esophagogastric junction; DCI: Distal contractile integral.

**Figure 3:**
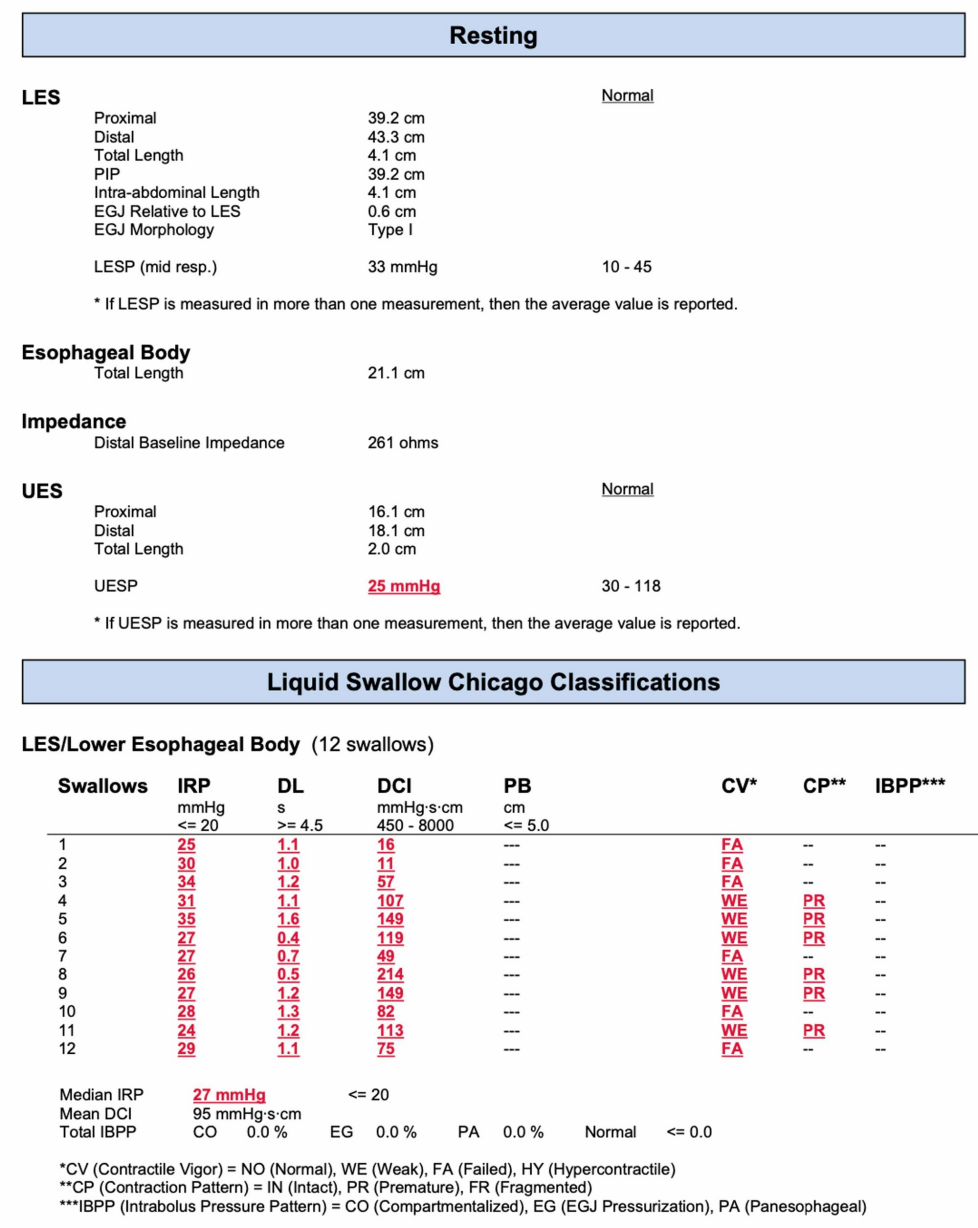
Manometry report.

**Figure 4:**
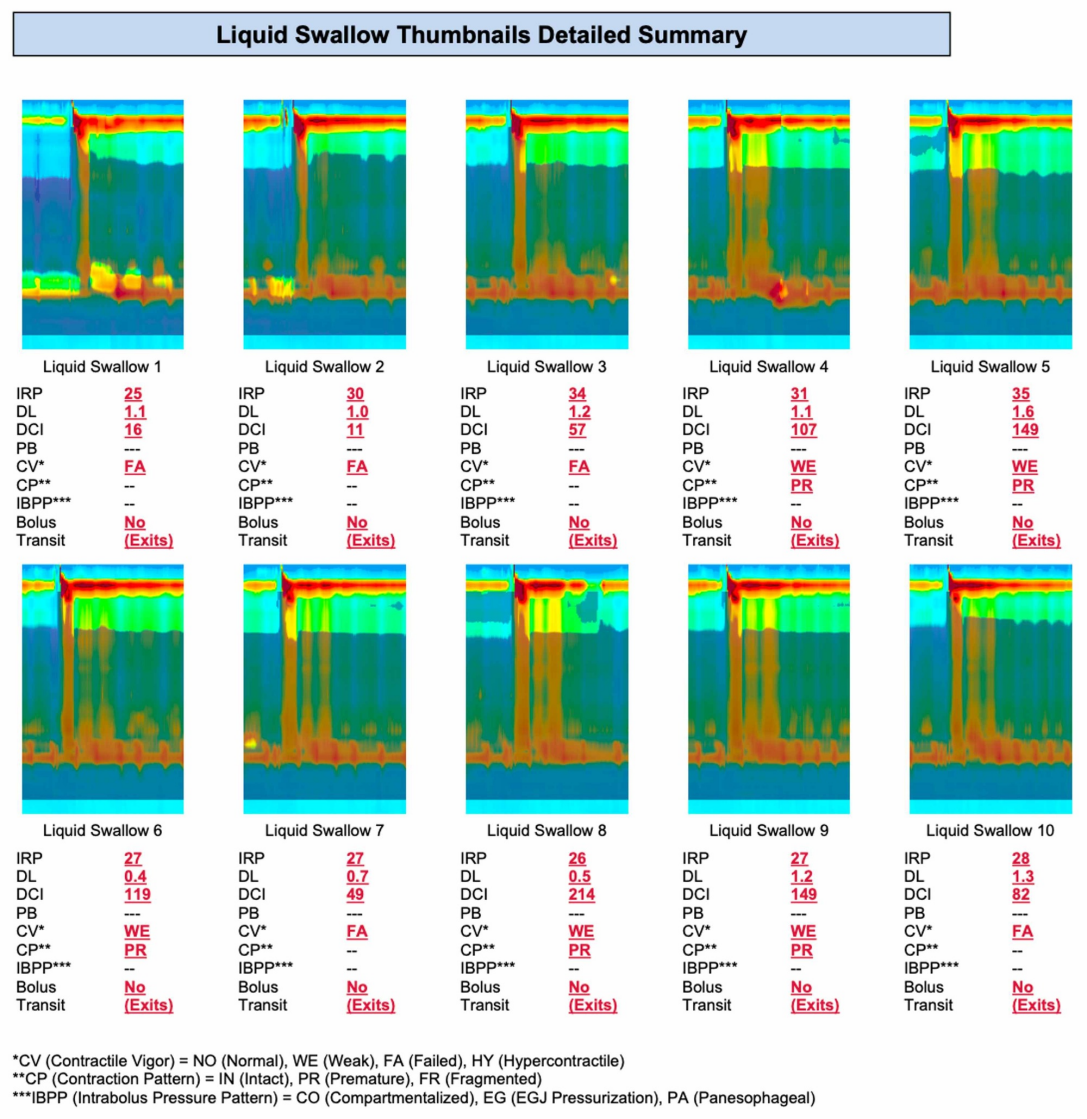
Manometry report.

The endoscope was removed. One hour later, manometry was performed, showing findings consistent with type II achalasia (Figures 2-4).

The inspection of the catheter revealed no evidence of damage to the catheter. The patient was discharged to follow up with a gastroenterologist as an outpatient for further management. She underwent endoscopic-guided botulinum toxin injection with temporary relief for two weeks. Peroral endoscopic myotomy (POEM) was performed one month later by advanced endoscopist without any complications with an improvement of dysphagia.

**Figure 5:**
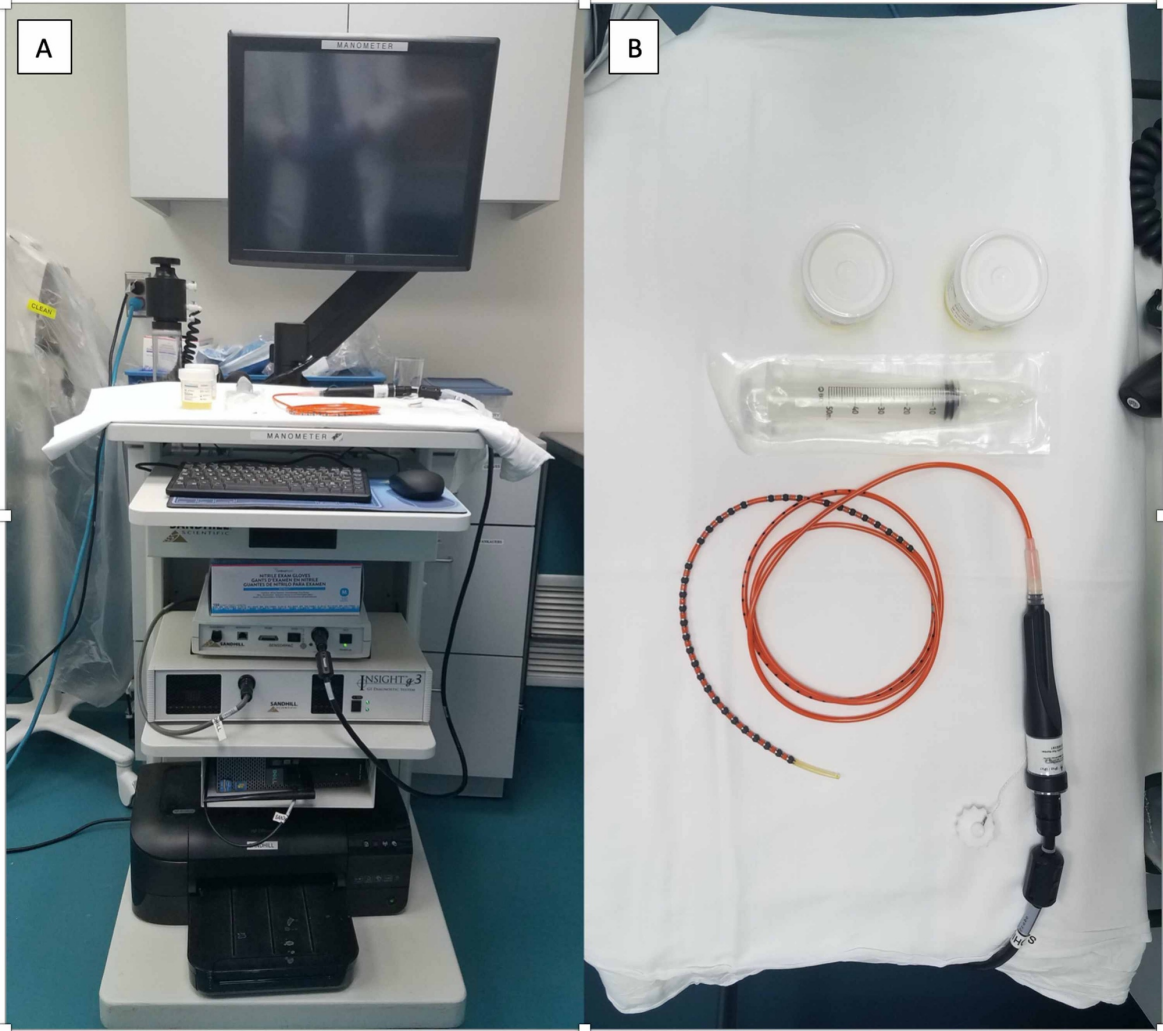
High resolution manometry set up (A) with manometry catheter (B).

**Figure 6:**
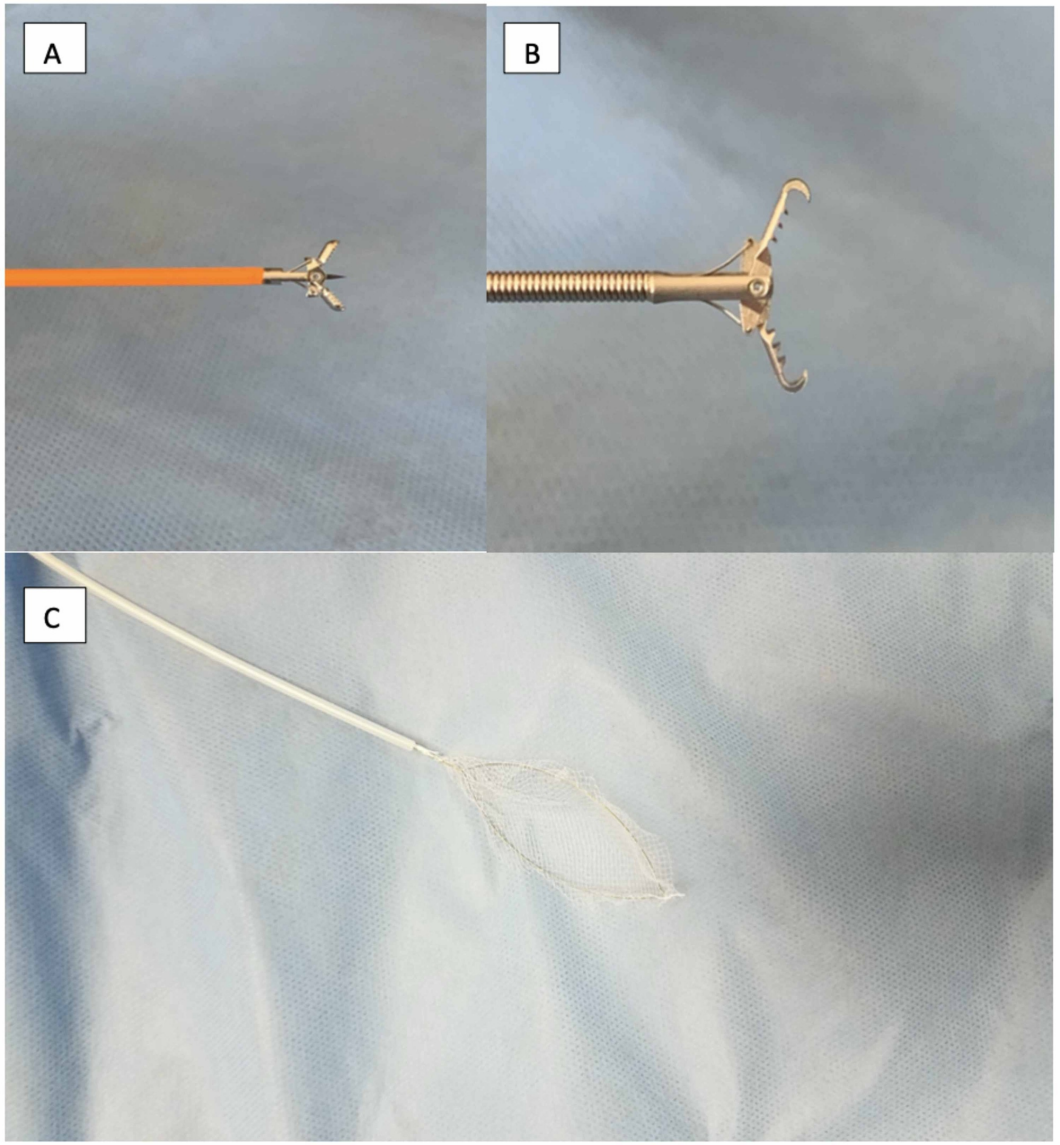
Biopsy forceps (A), rat tooth forceps (B) displaying sharp interdigitated teeth could be a potential damage to the manometry catheter. (C) Roth Net.

Pictures of motility set up, manometry catheter, endoscopic accessory devices are shown in Figure 5 and Figure 6, respectively.

## Discussion

Esophageal manometry to assess esophageal motility patterns is the standard test to diagnose esophageal motility disorders. Indications for performing the test include non-obstructive dysphagia, peristaltic reserve prior to anti-reflux surgery, symptoms of regurgitation, and non-cardiac chest pain [4]. The first conventional manometry system was developed by Wyle Jerry Dodds and Ron Arndorfer in 1970 [5], which has been state of the art for two decades till 1990. In 1990 Ray Clouse and his colleagues invented the HRM, which had revolutionized the clinical evaluation of esophageal motility disorders. Differences between conventional manometry and HRM are mentioned in Table 1. HRM evaluates the dynamic action of the upper esophageal sphincter, esophageal peristaltic patterns, and functional anatomy of GEJ. This data can be converted into seamless and dynamic spatiotemporal EPT or “Clouse plots” by advanced software algorithms. Measurements obtained from EPT plots like integrated relaxation pressure (IRP), distal contractile integral (DCI), distal latency (DL), and the contractile front velocity (CFV) can be used to classify esophageal motility disorder based on Chicago Classification (CC).

**Table 1:** Differences between conventional manometry and HRM HRM: High-resolution esophageal manometry; EPT: Esophageal pressure topography.

Conventional manometry	High resolution manometry
Cheap and durable	Expensive
Typically, 3-5 sensors wide apart [5]	Closely placed pressure sensors up to 36 in number 1 cm apart [5]
Low fidelity [5]	High fidelity [5]
Takes more time than HRM average time of 24.4 minutes [6,7]	Quick and easy placement of catheter average time of 8.2 minutes [6,7]
Need to reposition the catheter (pull through technique) [6]	No need for repositioning [6]
Uses water perfused catheters which are stiffer and more uncomfortable	Uses solid catheters which are softer and more comfortable
Unidirectional conventional line plot on the monitor [8,9]	Seamless and dynamic spatiotemporal EPT plots by advanced software algorithms [8,9]
Wave forms only [5]	Color contour [5]

HRM with EPT is a new technique that facilitates more accurate measurement of pressure changes in the esophagus by using a specialized catheter with very closely placed pressure censors 1 cm apart along the length of the catheter. The patient is asked to fast for at least 6 hours before the procedure and avoid medications that can alter esophageal motility on the day of the examination like calcium channel blockers, nitrates, prokinetics, loperamide, beta-receptor antagonists, opiates, and anticholinergics [10]. During the procedure, a manometry catheter is introduced trans-nasally and advanced into the esophagus and stomach in an awake patient, and the distal sensor is advanced 2-3 cm below the diaphragm and the LES is visualized in the Clouse view 2-3 cm below the diaphragm [11]. This procedure is usually performed without sedation, but several anatomical factors such as the deviated septum, prior craniofacial surgeries, esophageal tortuosity, and tight LES make it difficult. Endoscopy-assisted placement of the catheter can overcome these obstacles and facilitate safe placement, regrettably, endoscopic manipulation of the catheter can also damage the catheter and its sensors, rendering it unusable. The cost of the catheter approaches $19000, and once damaged, it cannot be repaired. Using a Roth Net inserted via the suction channel is a safe alternative in such situations to assist in the successful placement of the catheter, as in our case.

## Conclusions

In conclusion, using endoscopic accessories such as a Roth Net can be helpful for the successful placement of endoscopic-guided manometry catheter placement in cases where the procedure would otherwise be impossible without risking damage to the manometry catheter. We propose the use of Roth Net for safe and effective placement of the catheter, which can reduce the procedure time, necessary anesthesia, and related complications.
